# Responses of Macrobenthic Communities to Heavy Metal Contamination in Sediments and Seawater: A Case Study in Temperate Bay, South Korea

**DOI:** 10.3390/biology14091276

**Published:** 2025-09-16

**Authors:** Jian Liang, Se-Hyun Choi, Chae-Woo Ma

**Affiliations:** 1 Department of Food Science and Engineering, Xinjiang Institute of Technology, Aksu 843000, China; 20217196@sch.ac.kr; 2 Experimental Teaching Demonstration Center of Food Safety and Nutrition, Xinjiang Institute of Technology, Aksu 843000, China; 3 Aksu Institute of Apple, Xinjiang Institute of Technology, Aksu 843000, China; 4 Fisheries Business Team, Korea Fisheries Infrastructure Public Agency, Seoul 08588, Republic of Korea; dart8723@daum.net; 5 Department of Biology, College of Natural Sciences, Soonchunhyang University, Asan 31538, Republic of Korea

**Keywords:** Asan Bay, macrobenthos, Echinodermata, heavy metal, human activities

## Abstract

Although effects of heavy metals in sediments on macrobenthic organisms have been extensively studied, few investigations have simultaneously assessed the influence of heavy metals in seawater and sediments on macrobenthic communities. This study investigated subtidal macrobenthic communities of Asan Bay, a coastal area in South Korea subject to extensive anthropogenic disturbances. It aimed to assess sources of heavy metals in seawater and sediments and evaluate their influence on the structure and composition of macrobenthic communities. Our findings suggest that cadmium (Cd), chromium (Cr), arsenic (As), and mercury (Hg) in seawater mainly originate from terrestrial inputs. Although the concentrations of heavy metals in seawater and sediments at most sampling stations complied with the Korean Environmental Quality Standards, the results of redundancy analysis (RDA), Spearman correlation, distance-based linear models (DistLM), and distance-based redundancy analysis (dbRDA) indicated that these heavy metals still influenced the structure of macrobenthic communities. Heavy metals in seawater were found to have a greater impact than metals in sediments, with Cr in seawater exerting the most decisive influence on macrobenthos (RDA showed that Cr explained 47.6% of the constrained species–environment relationship). Thus, Cr concentrations in bay seawaters should receive attention. Existing seawater quality standards may need to be reconsidered to reflect their effects on macrobenthic communities better.

## Simple Summary

Coastal bays provide important habitats for marine life but are increasingly threatened by pollution from human activities. In this study, we investigated the macrobenthic communities of Asan Bay, South Korea, and examined how heavy metals in seawater and sediments influence their structure. Our results showed that, even though metal concentrations were within Korean environmental standards, they still had noticeable effects on benthic organisms. Metals in seawater were more harmful than those in sediments, and chromium in seawater had the strongest influence on community structure. These findings suggest that current water quality standards may not adequately protect benthic ecosystems. Special attention should be given to chromium reduction, and more integrated management is needed.

## 1. Introduction

Bays represent a unique type of coastal ecosystem, characterized by their semi-enclosed geomorphology, restricted water exchange with the open sea, and strong influence from terrestrial inputs. These features make bays more susceptible to anthropogenic disturbances while simultaneously supporting high habitat diversity, including spawning grounds, nursery areas, and feeding habitats that are critical for sustaining coastal biodiversity [1,2,3]. Globally, ecologically and economically important bays such as Chesapeake Bay, San Francisco Bay, Tokyo Bay (Japan), and the Bay of Bengal illustrate their vital roles in supporting biodiversity, fisheries, and human livelihoods. At the same time, bays are among the most vulnerable coastal systems to pollution, eutrophication, and habitat modification [4]. However, with the accelerating pace of urbanization and industrialization, the bay ecosystem has come under increasing pressure from multiple anthropogenic stressors [5,6,7]. Among these, heavy metal pollution stands out as one of the most critical threats, posing significant risks to marine organisms due to toxicity, persistence, and potential for bioaccumulation of these contaminants in the environment [8,9,10].

Macrobenthos, referring to benthic organisms larger than 0.5 mm in body size [11], play a vital role in marine ecosystems. They contribute to nutrient cycling and sediment stability. They also serve as key food sources for higher trophic levels [12,13]. Moreover, due to their relatively long lifespans, limited mobility, and high species diversity, macrobenthos exhibit various feeding strategies and varying responses to anthropogenic pressures [14,15]. These characteristics make them widely recognized as effective bioindicators for assessing anthropogenic pressures in marine ecosystems, as changes in their species richness, abundance, community composition, and functional traits are strongly linked to environmental disturbances [16,17].

Among various global environmental threats, heavy metals are regarded as one of the most critical due to their toxicity, persistence, and potential for bioaccumulation in ecosystems [18]. Previous studies have documented the effects of heavy metal pollution in sediments on macrobenthic species, taxa, community structure, and functional traits [19,20,21]. For instance, in Laoshan Bay, sedimentary heavy metal pollution has been shown to alter the trait composition and functional diversity of macrobenthic communities [19]. Similarly, research in Asan Bay has demonstrated that sediment-associated heavy metals significantly shape the structure of macrobenthic assemblages [22]. Despite these findings, investigations addressing the combined influence of heavy metals in both seawater and sediments on macrobenthic communities remain insufficient. This distinction is important because heavy metals in different environmental compartments affect macrobenthos in different ways. Metals dissolved in seawater are more readily absorbed by epifaunal species through respiration and filter feeding [23], whereas those bound to sediments mainly impact infaunal species via direct contact or ingestion [24,25]. Therefore, considering both seawater and sedimentary heavy metals provides a more comprehensive understanding of their ecological impacts on macrobenthic communities in coastal ecosystems

This study focuses on Asan Bay, a temperate embayment on the west coast of South Korea that has been subjected to multiple anthropogenic pressures. The objectives were to (1) assess sources of heavy metals in seawater and sediments, (2) evaluate the structure of macrobenthic communities, and (3) investigate the relationship between macrobenthic community changes and heavy metal contamination.

## 2. Materials and Methods

### 2.1. Study Area

**Figure 1:**
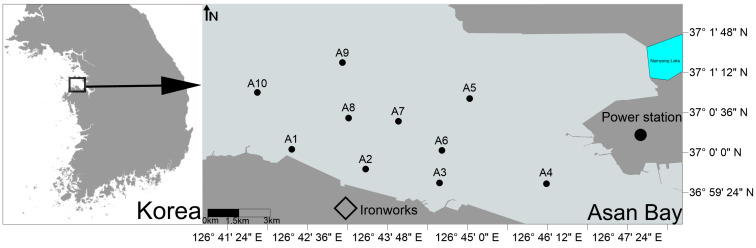
Sampling stations and study area in Asan Bay, South Korea.

Asan Bay is located in the northern part of the western coast of South Korea (Figure 1). It extends approximately 20 km from east to west and about 5 km from north to south, covering an area of around 80 km^2^. The bay hosts major industrial complexes and port facilities along both its northern and southern shores, serving as a key hub for South Korea’s economic infrastructure, particularly in manufacturing, shipping, and trade [26]. Since the 1970s, large-scale land reclamation projects have significantly reduced the bay’s water surface area, subjecting the ecosystem to multiple pressures from urbanization and industrialization. Precipitation in this region is concentrated between June and September each year, with more than 200 million tons of freshwater discharged annually from six nearby artificial lakes, shaping Asan Bay as a typical temperate embayment. The specific study area was located in the northern part of the bay, near a gas-fired power plant and an ironworks facility (Figure 1).

### 2.2. Sampling Collection

Sampling was conducted at ten stations during spring tide periods in May, August, and November of 2010 and in February, June, and September of 2011 (Figure 1). Sampling was conducted at all ten stations during each of the six survey periods. These months were selected to represent seasonal conditions. For operational consistency, sampling was scheduled during spring-tide periods. At the sampling stations, water depth ranged from 8 to 20 m, the distance from shore ranged from 0.5 to 4 km, and the substrate type varied from fine sand to clay. Geographic coordinates of these ten sampling stations are provided in Table S1. Bottom seawater samples were collected using a Niskin sampler. Parameters of the bottom seawater, including temperature, salinity, pH, and dissolved oxygen (DO), were measured onboard using a YSI-6920 multiparameter probe. The remaining seawater samples were stored in a deep freezer and transported to the laboratory for further analysis.

A 0.1 m^2^ van Veen grab was used to collect macrobenthic and sediment samples. At each station, two macrobenthic samples were taken, yielding a total sampling area of 0.2 m^2^, while sediment samples were collected once per station (0.1 m^2^). On board, macrobenthic samples were sieved through a 0.5 mm mesh to retain organisms. They were preserved in 5% neutral buffered formalin. Sediment samples were stored in a deep freezer and transported to the laboratory for subsequent analysis.

### 2.3. Sample Analysis

#### 2.3.1. Heavy Metal Analysis

To evaluate concentrations of heavy metals in sediments, samples were placed in 60 mL Teflon digestion vessels and digested on a hot plate using a mixed acid solution (HF + HNO_3_ + HClO_4_, 10 mL). After complete digestion and evaporation, residues were re-dissolved in 1% HNO_3_. Concentrations of trace metals (As, Cd, Cr, Cu, Pb, and Zn) were determined using an inductively coupled plasma mass spectrometer (ICP-MS; X-5, Thermo Elemental Ltd., Winsford, UK).

For seawater samples, dissolved trace metals were analyzed after on-site filtration through pre-cleaned 0.45 μm membrane filters (trace-metal-grade, acid-washed) using a peristaltic pump system in a clean environment to minimize contamination. Filtrates were immediately acidified to pH < 2 with ultrapure HNO_3_ (Seastar Chemicals, Sidney, BC, Canada, trace-metal grade). The pH of acidified samples was measured and consistently below 2.0. All containers, filters, and tubing were pre-cleaned with dilute HNO_3_ and rinsed with ultrapure water prior to use. Acidified samples were stored in trace-metal-clean polyethylene bottles at 4 °C until analysis. Concentrations of dissolved metals (As, Cd, Cr, Cu, Pb, and Zn) were measured using the same ICP-MS instrument. Chromium was measured as total Cr due to methodological limitations, without distinguishing between Cr (III) and Cr (VI), although we acknowledge that their ecotoxicological behaviors differ markedly. Similarly, total Hg was measured by CVAAS, and the more toxic methylmercury fraction was not speciated. Total mercury (Hg) concentrations in seawater and sediment samples were determined using cold vapor atomic absorption spectrophotometry (CVAAS; Model 400A, BUCK Scientific, East Norwalk, CT, USA). Prior to digestion, the moisture content of each sediment sample was determined gravimetrically by oven-drying subsamples at 105 °C until constant weight. All metal concentrations were expressed on a dry-weight basis. In addition, to minimize the influence of grain-size heterogeneity on trace metal comparisons, the <63 μm fine fraction was isolated by wet sieving after removal of organic matter with hydrogen peroxide (H_2_O_2_).

#### 2.3.2. Nutrient and Oxygen Demand Analysis

For total nitrogen (TN), samples were digested using alkaline persulfate oxidation and subsequently analyzed by ultraviolet spectrophotometry (Shimadzu UV-1800, Kyoto, Japan) at 220/275 nm. For total phosphorus (TP), samples were digested with potassium persulfate and analyzed using the molybdenum blue colorimetric method with absorbance measured at 880 nm. Calibration was performed using potassium nitrate and potassium dihydrogen phosphate standards, respectively. Chemical oxygen demand (COD) in seawater and sediment was measured using the titrimetric method based on potassium dichromate oxidation [27]. Acid volatile sulfide (AVS) concentrations in sediments were determined using a detection tube method [27].

#### 2.3.3. Suspended Solids and Organic Matter Analysis

Suspended solids in seawater were quantified by filtering samples through pre-weighed 0.45 μm glass fiber filters, followed by drying at 105 °C to constant weight. Ignition loss (IL) of sediments, representing organic matter content, was determined by heating pre-dried samples at 550 °C for 2 h in a muffle furnace.

#### 2.3.4. Particle Size Analysis

Before grain size analysis, sediment samples were pretreated with hydrogen peroxide (H_2_O_2_) to remove organic matter. Particles larger than 63 μm were analyzed using a standard dry sieving method, while particles smaller than 63 μm were separated by wet sieving. Grain size distribution of the fine fraction was subsequently measured using a laser diffraction particle size analyzer (Sympatec GmbH, Clausthal-Zellerfeld, Germany, HELOS/RODOS model).

#### 2.3.5. Macrobenthic Identification

In the laboratory, macrobenthic organisms were identified to the lowest possible taxonomic level under a dissecting microscope SMZ-168 (Motic, Xiamen, China). All individuals were counted. Specimens were subsequently transferred into labeled glass jars and preserved in 70% ethanol.

#### 2.3.6. Quality Control

All analytical procedures for seawater and sediment samples followed protocols outlined in the Notification of Marine Environmental Process Test Standards issued by the National Institute of Fisheries Science (2010) [27]. To ensure analytical accuracy and reproducibility, quality assurance and quality control (QA/QC) procedures were strictly followed. Limits of detection (LOD) and quantification (LOQ) for each element were determined as three and ten times the standard deviation of procedural blanks, respectively. Method blanks, spike/recovery tests (85–110%), and certified reference materials (NIST SRM 1646a Estuarine sediment) were included in each analytical batch. The relative standard deviation (RSD) of replicate analyses was generally <5%. Each analysis was conducted in triplicate, and deviations beyond the accepted QC range were reanalyzed.

### 2.4. Data Analysis

#### 2.4.1. Dominance and Diversity Indices

A dominance index was calculated to identify dominant species within the macrobenthic community of Asan Bay, with species exhibiting dominance values greater than 0.02 classified as dominant [28]. To evaluate macrobenthic diversity, four diversity indices (Species richness index, Pielou’s evenness index, Simpson index, and Shannon-Wiener diversity index) were employed. Their respective formulas are detailed in Table S2.

#### 2.4.2. Analysis of Community Structure

Before analysis, macrobenthic community composition data (species identity and abundance matrix) were log(x+1)-transformed. Community clustering (using hierarchical agglomerative clustering with group-average linkage based on Bray–Curtis similarity) and non-metric multidimensional scaling (NMDS) were conducted in PRIMER version 7 (PRIMER-e, New Zealand) to assess monthly variations in macrobenthic communities in Asan Bay.

#### 2.4.3. Statistical Analysis

A series of multivariate statistical analyses was performed to investigate the influence of environmental factors on the macrobenthic community in Asan Bay. Principal Component Analysis (PCA) was conducted in PRIMER version 7 (PRIMER-e, New Zealand) to reduce the dimensionality of the environmental dataset and identify key gradients explaining the most significant variance.

Spearman correlation heatmaps were generated in R version 4.3.2 (packages psych and ggplot2) to visualize pairwise associations between environmental variables and macrobenthic community metrics. Hierarchical clustering based on correlation distance and average linkage was applied to group variables with similar association patterns. To account for multiple testing, the Benjamini–Hochberg false discovery rate (BH-FDR) correction was applied, and correlations were considered significant at an adjusted *p* < 0.05.

Given the sparsity and large variation in macrobenthic community abundances, the raw abundance data were log(x+1)-transformed prior to multivariate analyses to reduce the influence of highly dominant taxa. Environmental variables were standardized to zero mean and unit variance to eliminate the effects of differing measurement scales.

To diagnose collinearity among environmental variables, variance inflation factor (VIF) values were calculated in R 4.3.2 using the usdm package. Variables with VIF > 10 or pairwise correlations |r| > 0.8 were considered collinear; in such cases, the variable with clearer ecological relevance was retained while the redundant one was removed. This procedure excluded several highly collinear variables (e.g., salinity, pH, DO, TN, TP, COD), and a final subset of nine predictors (Seawater temperature, Pb in seawater, Cr in seawater, Cu in seawater, As in seawater, Hg in seawater, ignition loss, Cr in sediment, and Hg in sediment) was retained for subsequent analyses.

A distance-based linear model (DistLM) was employed to identify the most influential environmental drivers of community structure. Results were visualized using distance-based redundancy analysis (dbRDA), based on Bray–Curtis dissimilarity of log(x+1)-transformed abundance data, providing a constrained ordination of macrobenthic assemblages according to selected predictors. The BIO-ENV procedure was applied to determine the best subset of variables explaining biological patterns. DistLM and dbRDA significance levels were assessed using 999 permutations.

In addition, two-way permutational multivariate analysis of variance (PERMANOVA) was used to test for significant spatiotemporal differences in community structure, using Bray–Curtis dissimilarity as the resemblance metric and 999 permutations for significance testing. PERMANOVA was also applied to environmental variables to assess their spatial differences among sampling stations. To complement these analyses, classical Redundancy Analysis (RDA) was performed using CANOCO version 5.0 (Biometris–Plant Research International, Wageningen, The Netherlands) to further evaluate species–environment relationships. In the RDA, dominant species were retained in order to reduce background noise and highlight the most representative taxa. All statistical analyses were performed using PRIMER version 7 (PRIMER-e, Wellington, New Zealand), CANOCO version 5.0, and R version 4.3.2 (packages vegan, usdm, ggplot2, and psych).

## 3. Results

### 3.1. Environment Data Characteristics

Environmental data for seawater and sediments in the subtidal zones of Asan Bay are presented in Table 1 and Table 2. Among seawater parameters, chromium (Cr) exhibited the highest coefficient of variation (CV) (0.80), while pH had the lowest CV (0.01). For sediment parameters, chemical oxygen demand (COD) showed the highest CV (0.45), whereas cadmium (Cd) had the lowest CV (0.15). Average concentrations of heavy metals in seawater showed the following order: Hg < Cr < Cd < As < Pb < Cu < Zn. Those in sediments showed the following order: Hg < As < Cd < Cu < Cr < Pb < Zn.

**Figure 2:**
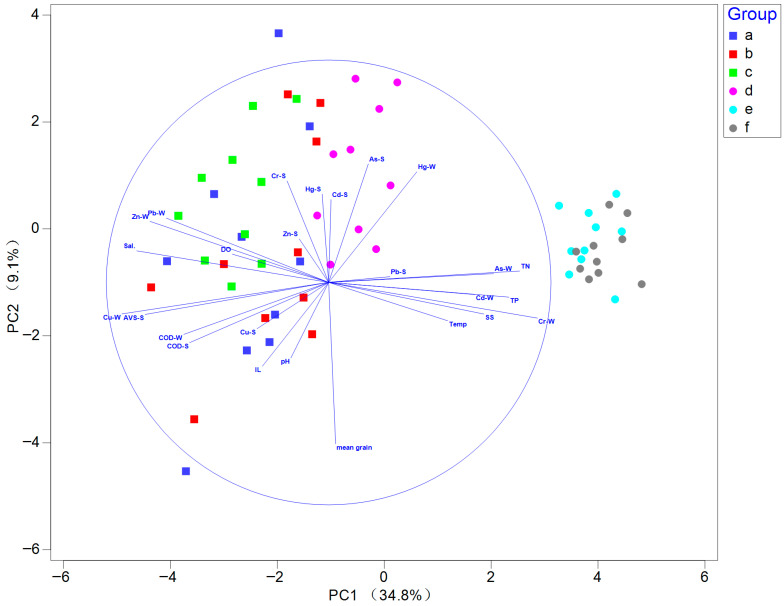
Principal component analysis (PCA) plot of environmental parameters in Asan Bay. Note: (a–c) represent sampling periods in May, August, and November 2010, respectively; (d–f) represent February, June, and September 2011, respectively. S indicates sediment samples, and W indicates seawater samples. Abbreviations: AVS, acid-volatile sulfide; COD, chemical oxygen demand; DO, dissolved oxygen; IL, ignition loss. The blue circle indicates the correlation circle, representing the maximum correlation of variables with the first two principal components. Blue arrows denote environmental variables, with direction indicating correlation and length representing contribution strength.

In the principal component analysis (PCA), the first two components explained a cumulative variance of 43.9%, with the first and second axes accounting for 34.8% and 9.1% of the cumulative variance, respectively (Figure 2). Sampling stations from June (e) and September (f) 2011 were clearly separated from the others, suggesting that seawater samples during these sampling periods contained relatively higher concentrations of Cr, total nitrogen, and total phosphorus than those collated at other sampling periods (Tables S3 and S4). PERMANOVA results indicated that environmental variables exhibited significant temporal differences across sampling months (F = 10.1; *p* < 0.001) and years (F = 20.16; *p* < 0.001).

### 3.2. Macrobenthic Composition and Community Structure

**Figure 3:**
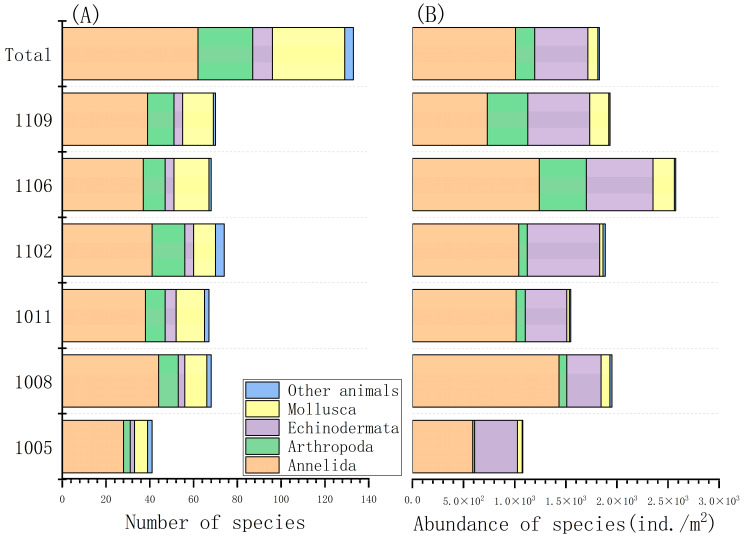
Composition and abundance of macrobenthic communities in the subtidal zones of Asan Bay. Note: Sampling codes on the y-axis represent the year and month of sampling (e.g., 1005 = May 2010). (**A**) Number of species; (**B**) Abundance of species.

A total of 133 macrobenthic species were identified across the six sampling periods. These included 62 species of Annelida, 33 species of Mollusca, 25 species of Arthropoda, 9 species of Echinodermata, and 4 species classified as other animals. The highest species richness was observed in February 2011 with 74 species, while the lowest was recorded in May 2010 with 41 species (Figure 3A). The overall mean abundance of macrobenthic organisms showed 1828.3 ± 495.9 ind./m^2^, ranging from a minimum of 1078 ind./m^2^ in May 2010 to a maximum of 2576 ind./m^2^ in June 2011 (Figure 3B). Based on the dominance index, four macrobenthic species were classified as dominant: *Amphiodia craterodmeta* (0.20), *Heteromastus filiformis* (0.17), *Ampharete finmarchica* (0.10), and *Corophium* sp. (0.03) (Table 3).

**Figure 4:**
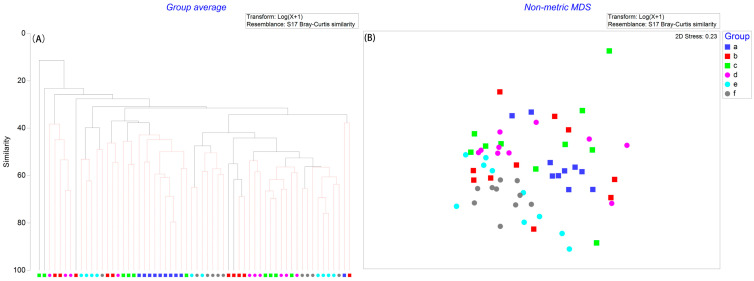
Cluster analysis (**A**) and non-metric multidimensional scaling (NMDS) plots (**B**) based on the Bray–Curtis similarity of the abundance of macrobenthos in subtidal zones of Asan Bay. Note: (a–c) represent sampling periods in May, August, and November 2010, respectively; (d–f) represent February, June, and September 2011, respectively.

Cluster analysis (A) and NMDS (B) plots revealed a clear separation of sampling stations among months (Figure 4), whereas no distinct separation was observed among years. This pattern was further supported by PERMANOVA results, which indicated significant differences in macrobenthic community composition among months (F = 3.60, *p* = 0.021), but not among years (F = 4.03, *p* = 0.061).

### 3.3. Results of Diversity Indices

For the four diversity indices, the average values was 2.94 for the species richness index (d), 0.69 for Pielou’s evenness index (J′), 0.75 for Simpson’s diversity index (1–Lambda′), and 2.97 for the Shannon–Wiener diversity index (H′). Values of these four diversity indices at each station are shown in Figures S1–S4.

### 3.4. Relationship Between Macrobenthos and Environmental Factors

**Figure 5:**
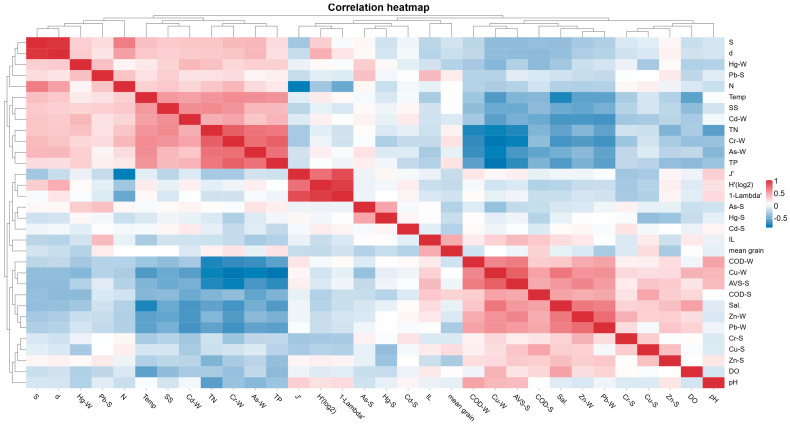
Spearman correlation heatmap between Species richness, abundance, Four Diversity Indices, and Environmental Variables. Note: N, Number of species; A, Abundance of species; d, Species richness index; J’, Pielou’s evenness index; H’, Shannon–Wiener diversity index; 1-Lambda’, Simpson’s diversity index; AVS, acid-volatile sulfide; COD, chemical oxygen demand; DO, dissolved oxygen; IL, ignition loss; Temp, Seawater temperature; TP, Total Phosphorus; TN, Total Nitrogen; SS, Suspended Solids; Mean, Mean grain size. S indicates sediment samples, and W indicates seawater samples; The color scale represents correlation strength and direction. Blue indicates negative correlations and red indicates positive correlations. Significance levels were adjusted using the Benjamini–Hochberg false discovery rate (BH-FDR).

Results of Spearman correlation analysis indicated that the number of species was significantly correlated with TN, SS, and heavy metals in seawater, but only correlated with AVS or COD in sediments. The abundance of species showed correlations with TN, SS, Cd, Cr, and Cu in seawater and Pb in sediments. Species richness index was correlated with seawater temperature, salinity, TN, SS, Zn, Cd, Cr, Cu, As, and Hg, and AVS and COD in sediments. Pielou’s evenness index correlated with seawater pH, Cr, and Cr and Cu in sediments. The Shannon–Wiener diversity index was correlated with Cr in sediments. Simpson’s diversity index (1-Lambda’) was also correlated with sediment Cr (*p* < 0.05) (Figure 5).

DistLM results indicated that IL and sediment concentrations of Cr and Hg did not significantly (*p* > 0.05) contribute to the variation in macrobenthic community composition (Table 4).

**Figure 6:**
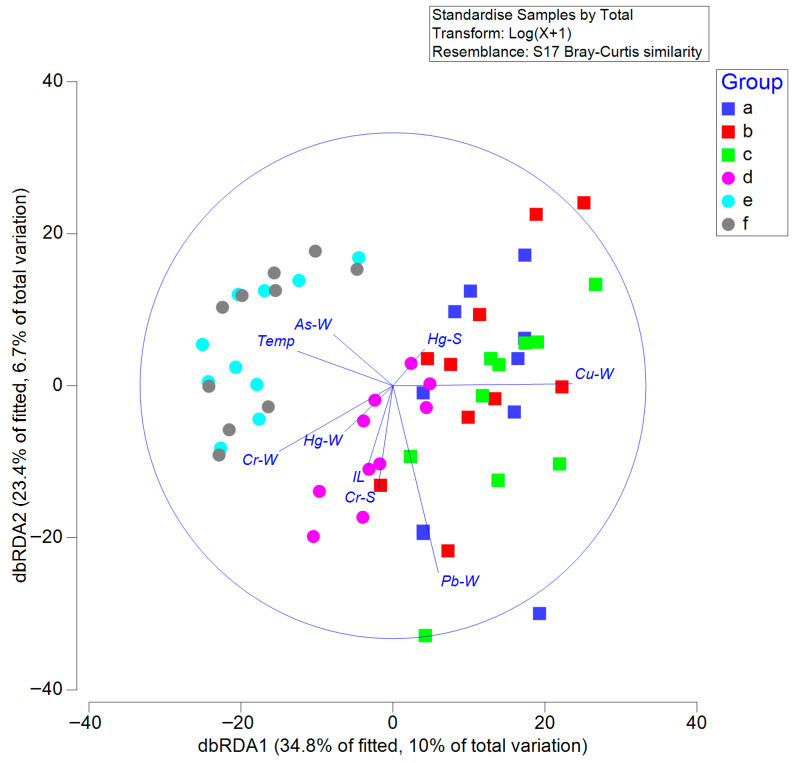
Distance-based redundancy analysis (dbRDA) ordination of macrobenthic community composition constrained by nine environmental variables. Note: (a–c) represent sampling periods in May, August, and November 2010, respectively; (d–f) represent February, June, and September 2011, respectively. Temp, seawater temperature. S indicates sediment samples and W indicates seawater samples. Statistical significance was assessed using permutation tests (999 permutations, *p* < 0.01).

In the dbRDA plot, axes 1 and 2 together explained 58.2% of the constrained variance, which corresponded to 16.7% of the total variance in the data (Figure 6). The overall dbRDA model explained 28.6% of the total variance and was statistically significant (999 permutations, *p* < 0.01). Among the tested variables, Cr concentration in seawater contributed most strongly to the separation along dbRDA axes 1 and 2 (Table S5). Sampling stations from June (e) and September (f) 2011 were clearly separated from those of other months, clustering in the lower right quadrant of the plot (Figure 6). This suggests that elevated Cr concentrations in seawater influenced the macrobenthic community structure during these periods.

**Figure 7:**
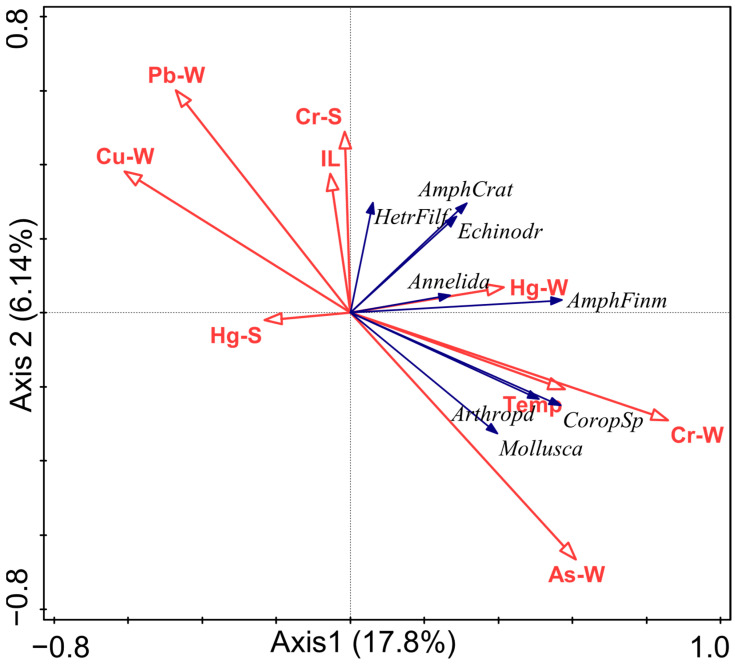
Redundancy analysis (RDA) results of the relationship between macrobenthos and Environmental Variables. Note: IL, ignition loss; Temp, Seawater temperature; Arthropd, Arthropoda; Echinodr, Echinodermata; AmphFinm, *Ampharete finmarchica*; AmphCrat, *Amphiodia craterodmeta*. CoropSP, *Corophium* sp., HetrFilf, *Heteromastus filiformis*. S indicates sediment samples and W indicates seawater samples.

In the RDA, Axis 1 and Axis 2 together explained 23.94% of the constrained variance, with Axis 1 accounting for 17.8% and Axis 2 for 6.14% of the variance (Figure 7). Among environmental variables, Cr in seawater was significantly correlated with the spatiotemporal distribution of macrobenthic communities (*p* < 0.05), accounting for 47.6% (Table S6).

The BIO-ENV analysis of environmental variables and macrobenthic abundance data indicated that the combination of seawater temperature with Pb, Cr, and Cu in seawater represented the best explanatory set, contributing most to the observed community patterns (Table S7).

## 4. Discussion

### 4.1. Heavy Metal Characteristics and Sources in Asan Bay

In our study, average concentrations of six heavy metals in the seawater of Asan Bay were higher than those observed in coastal waters of Jeju Island and Jindo Island, areas characterized by relatively low anthropogenic activities. However, compared to the highly industrialized Gyeonggi Bay and Masan Bay, Cd levels in Asan Bay were higher than those in Masan Bay, while Cu, Pb, and Zn concentrations in Asan Bay were higher than those in both Gyeonggi Bay and Masan Bay. Despite these differences, the mean concentration of Cu in seawater and the Pb concentrations at some stations in Asan Bay exceeded the limits of the Korean marine seawater quality standard. (Table 5). Average concentrations of Cd and Pb in sediments of Asan Bay were higher than those recorded in the East Sea of Korea, whereas average concentrations of four heavy metals (Cd, Cu, Pb, and Zn) in sediments of Asan Bay were lower than those found in Korea’s highly industrialized South Sea. Asan Bay may be attributed to the presence of extensive industrial facilities, including steel plants and heavy manufacturing complexes, located along the bay. These land-based anthropogenic inputs contrast with the central East Sea region, which lacks such intensive industrial development. Average concentrations of five heavy metals (As, Cd, Cu, Pb, and Zn) in sediments of Asan Bay were also lower than those reported in Dangdong Bay, an area with extensive shipbuilding activities. However, concentrations of heavy metals in sediments of Asan Bay remained below the Threshold Effect Level (TEL) and Probable Effect Level (PEL), indicating a limited ecological risk (Table 6). Geographic locations of the study area (Asan Bay) and comparison sites along the Korean coast are shown in Figures S5 and S6.

Spearman correlation analysis revealed distinct patterns in the distribution and potential sources of heavy metals in seawater and sediments (Figure 6). In seawater, Cd, Cr, As, and Hg showed significant positive correlations with TN, TP, and SS, but significant negative correlations with salinity (all *p* < 0.05). Toxic heavy metals such as Cd, Cr, As, and Hg are predominantly derived from anthropogenic activities, particularly industrial emissions, agricultural runoff, and urban effluents, known to have significantly intensified their presence in aquatic ecosystems [29,30]. This indicates that these metals are likely to be associated with land-based inputs. Cd in the seawater of Gyeonggi Bay, located in the northern part of Asan Bay, is likewise primarily attributed to terrestrial inputs [31]. In contrast, Zn, Pb, and Cu in seawater exhibited significant positive correlations with salinity but significant negative correlations with TN, TP, and SS. This suggests that Zn, Pb, and Cu are primarily influenced by marine water exchange rather than terrestrial inputs, potentially originating from natural background levels or offshore seawater intrusion (*p* < 0.05).

The distribution of heavy metals in the seawater column and sediments is interdependent and subject to change. Variations in hydrodynamic conditions and water quality can jointly regulate the mobility and transformation of these metals across environmental compartments [32,33]. Cu and Zn exhibited significant positive correlations with AVS and COD in sediments, while Pb showed a significant positive correlation with IL (all *p* < 0.05). These relationships suggest that these metals can accumulate under reducing conditions and that they are commonly associated with organic matter or sulfides. The significant positive correlation between Zn in seawater and Zn in sediments indicates a potential common source or exchange between these two compartments (*p* < 0.05). In contrast, the significant negative correlation between Cr in seawater and Cr in sediments suggests transformation or redistribution between the water column and sediments (*p* < 0.05). Cd in sediments showed no significant correlations with environmental variables, indicating that other factors might have influenced its distribution (*p* > 0.05). Additionally, As in sediments exhibited significant positive correlations with Hg in seawater and sediments as well as Pb in sediments (*p* < 0.05). This suggests that these metals are likely to share a common source. Furthermore, the steel plant in the southern part of Asan Bay is an additional source of heavy metal contamination through atmospheric deposition [34]. Several studies have demonstrated that steel manufacturing emissions can significantly increase concentrations of Cd, Zn, Cu, Pb, and Hg in the surrounding environment [35,36].

**Table 5:** Comparison of heavy metal concentrations (μg/L) and average concentrations in seawater between the study area and other areas.

Study Area	As	Cd	Cr	Cu	Pb	Zn	Hg	Reference
Asan Bay	0.13–0.89 (0.33)	0.13–0.44 (0.27)	0.05–0.68 (0.25)	0.36–4.30 (1.76)	0.16–1.94 (0.60)	0.76–6.46 (2.86)	0–0.01 (0.002)	This study
Jindo Island	0.040–0.200 (0.090)	0.004–0.070 (0.040)	0.020–0.160 (0.080)	0.120–0.500 (0.270)	0.010–0.070 (0.030)	0.120–0.570 (0.340)	NA	[37]
Jeju Island	0.020–0.050 (0.030)	0.001–0.050 (0.020)	0.010–0.120 (0.040)	0–0.170 (0.100)	0.009–0.030 (0.020)	0.090–0.450 (0.260)	NA	[37]
Gyeonggi Bay, Korea	NA	0.037–0.073 (0.048)	NA	0.460–1.020 (0.610)	0.010–0.035 (0.020)	0.140–1.190 (0.360)	NA	[31]
Masan Bay, Korea	NA	0.007–0.027 (0.015)	NA	0.420–1.010 (0.660)	0.003–0.053 (0.012)	0.250–3.700 (1.420)	NA	[38]
Korea marine seawater quality standard	3.4	1.6	2.8	1.2	1.6	11	1.0	[39]

**Table 6:** Comparison of heavy metal concentrations (mg/kg) and average concentrations in sediments between the study area and other areas.

Study Area	As	Cd	Cr	Cu	Pb	Zn	Hg	Reference
Asan Bay	0.13–0.64 (0.35)	0.47–0.98 (0.71)	2.03–22.82 (11.39)	5.01–13.54 (9.02)	12.47–33.20 (21.71)	18.48–65.07 (46.12)	0–0.01 (0.003)	This study
East Sea of Korea	1.91–6.14 (3.35)	0.04–0.33 (0.124)	8.71–66.98 (43.7)	1.83–32.56 (16.6)	9.79–32.27 (20.48)	11.27–109.31 (65.9)	0.01–0.06 (0.022)	[40]
South Sea of Korea	NA	NA	6.80–165 (67.8)	4.7–100.4 (21.9)	11.5–91.4 (31.6)	22–312 (109)	NA	[41]
Dangdong Bay, Korea	0.30–0.70 (0.48)	1.40–3.30 (2.56)	NA	13.90–22.50 (17.17)	35.80–60.00 (50.06)	67.90–84.90 (76.10)	NA	[22]
Threshold Effect Level	14.50	0.75	116	20.6	44.00	64.40	0.11	[39]
Probable Effect Level	75.5	2.72	181	64.4	119	157	0.62	[39]

### 4.2. Macrobenthic Community in Asan Bay

Across the six sampling periods, Annelida exhibited the highest species richness with 62 species, followed by Mollusca with 33 species. Regarding the average abundance, Annelida also dominated, with an average of 1007.23 individuals/m^2^, while Echinodermata, despite having fewer species (9 species), ranked second in abundance with an average of 521.17 individuals/m^2^. In other regions along the west coast of Korea, such as Cheonsu Bay, Anmyeon Island, and Garolim Bay, similar patterns have been observed, with Annelida consistently exhibiting the highest abundance and species richness [42,43,44], consistent with findings of the present study.

*Amphiodia craterodmeta* was found to be the most abundant species in the macrobenthic communities along the Asan Bay in this study. This is a highly unusual finding that has been rarely reported in previous studies. *Amphiodia craterodmeta* was predominantly abundant along shipping lanes and ports, with particularly high densities recorded at Station 7 in May 2010 and Station 4 in June 2011, where its density exceeded 3000 individuals/m^2^ (http://www.khoa.go.kr/oceanmap/main.do# accessed on 1 January 2025). Dredging activities in shipping lanes and ports might have caused short-term disturbances and mortality among benthic organisms [45,46], which might have created favorable conditions for the opportunistic, detritivorous *Amphiodia craterodmeta* to increase in abundance temporarily [47]. Furthermore, in the RDA, *Amphiodia craterodmeta*, compared to other dominant species, exhibited a degree of tolerance to elevated levels of Cr (Figure 7). This association might reflect a greater resilience to such environmental conditions, supporting its proliferation in disturbed habitats. At the physiological level, echinoderms are known to synthesize metallothioneins and antioxidant enzymes, which can bind and detoxify Cr, thereby alleviating oxidative stress effects [48]. Such dredging-related disturbances may not only favor opportunistic species like *Amphiodia craterodmeta* but also reduce species richness and evenness in the overall community. This can lead to a decline in functional diversity and potentially disrupt key ecosystem processes such as sediment bioturbation, nutrient cycling, and habitat provision, thereby impairing the ecological resilience of Asan Bay.

In addition to spatial differences, the macrobenthic community exhibited clear temporal variation across the six sampling months from 2010 to 2011. This seasonal pattern is consistent with our previous findings from Jeju Island and Jindo Island, where benthic assemblages also showed pronounced seasonal fluctuations [37]. In Asan Bay, such temporal differences appear to be influenced by seasonal land-based inputs of Cr.

### 4.3. Influence of Heavy Metals on Macrobenthic Community

In this study, the concentrations of most heavy metals in seawater in Asan Bay complied with the national marine water quality standards of Korea, as defined by the Ministry of Oceans and Fisheries, with the exception of Pb and Cu in seawater (Table 5 and Table 6). However, results from RDA, Spearman correlation, DistLM, and dbRDA consistently demonstrated that heavy metals in seawater and sediments significantly influenced the macrobenthic community structure. Notably, the analysis revealed that, even at relatively low concentrations, heavy metals in seawater exerted a markedly greater impact on the macrobenthic community than those metals in sediments. Differences in metal bioavailability and exposure pathways might explain this disparity. Heavy metals dissolved in seawater are more readily bioavailable to macrobenthos, particularly epifaunal species, which are continuously exposed to heavy metals through respiration, filter feeding, and surface sediment ingestion [49,50]. In contrast, heavy metals in sediments are typically bound to particulate matter and organic substances such as iron, manganese, and aluminum oxides, clay minerals, and organic matter through processes including adsorption, chelation, and sorption, which can substantially reduce their bioavailability [51]. Under stable redox conditions, these metals remain largely immobilized in less bioavailable forms, limiting their uptake and toxicity to macrobenthic organisms [52]. In contrast, hydrodynamic processes and anthropogenic inputs can cause seasonal or episodic fluctuations in seawater metal concentrations, increasing the risk of acute exposure for benthic organisms and amplifying ecological impacts on sensitive species [53]. In particular, the dbRDA revealed that macrobenthic community structures in June and September 2011 differed markedly from those during other sampling periods. This variation is likely attributable to increased Cr inputs during the rainy season, when terrestrial runoff carries heavy metals from surrounding watersheds into Asan Bay.

The RDA and DistLM analyses identified Cr concentrations in seawater as a key environmental factor influencing the macrobenthic community structure in Asan Bay. Our previous study similarly demonstrated that Cr in seawater was also a critical factor shaping benthic communities along the coasts of Jeju Island and Jindo Island [37]. In aquatic environments, Cr primarily exists in two oxidation states: Cr (III) and Cr (VI), both forming soluble complex anions [54]. Cr inputs are primarily derived from industrial discharges and urban runoff, contributing to its accumulation in coastal waters [55]. Of these, Cr (VI) is more toxic and bioavailable than Cr (III) due to its higher solubility and oxidative potential, posing greater risks to marine organisms by impairing respiration, enzyme activity, and reproductive processes [56,57]. Ecologically, Cr (VI) is highly mobile and can enter organisms through sulfate transport pathways, causing oxidative stress and genotoxic effects [58]. In contrast, Cr (III) is less mobile and usually forms stable complexes with organic matter and minerals, which reduces its toxicity [59]. As a result, Cr (VI) poses a greater risk of acute impacts on species diversity and community structure, whereas Cr (III) is more associated with long-term accumulation and lower immediate effects [60].

A limitation of the present study is that chromium speciation was not measured, and only total Cr concentrations were analyzed. Consequently, we were unable to differentiate the specific ecological contributions of Cr (III) and Cr (VI) to the observed patterns in macrobenthic community structure. Future research should incorporate chromium speciation analyses to provide more precise assessments of ecological risks associated with chromium contamination in coastal ecosystems.

Effects of heavy metals on macrobenthos can differ according to the type of metal. Tolerance levels of different benthic species to the same heavy metal could also vary [61,62]. Consistent with this, the RDA in our study demonstrated that the four dominant species responded differently to various heavy metals (Figure 5).

### 4.4. Management Recommendations for Protecting the Bay

Since the 1960s, industrialization in South Korea has led to widespread anthropogenic impacts on bays along the west and south coasts, primarily due to industrial discharges, urban development, port construction, and aquaculture expansion [63,64]. Since the late 20th century, the Korean government has actively implemented marine environmental protection measures. However, coastal ecosystems remain under considerable pressure.

In this study, heavy metals in seawater, Cd, Cr, As, and Hg, were identified as predominantly land-based in origin. Although the concentration of Cr in seawater complied with the established water quality standards, findings suggest that Cr can exert adverse effects on macrobenthic communities even at it current levels. Thus, existing water quality standards might need to be re-evaluated and refined by incorporating ecological risk assessment frameworks to address this issue. Specifically, integrating sediment-based indices such as the TEF and PEF could allow for a more accurate assessment of cumulative metal toxicity and its ecological consequences on macrobenthos. In addition to revising standards, comprehensive management strategies should be implemented. These include strengthening the control of land-based pollution sources and establishing ecological buffer zones to intercept and reduce heavy metal inputs. Furthermore, integrating land-use policies, industrial regulations, and coastal zone management within an ecosystem-based management framework is critical for addressing land–sea ecological connectivity. For example, regulating agricultural runoff, enforcing stricter industrial discharge standards, and enhancing wastewater treatment facilities would help reduce terrestrial heavy metal inputs.

## 5. Conclusions

In our study, 133 macrobenthic species were recorded in the subtidal zone of Asan Bay, with annelids being the most abundant group. Although concentrations of heavy metals in both seawater and sediments met the Korea Marine Seawater Quality Standards, analyses using RDA, Spearman correlation, DistLM, and dbRDA showed that even at low concentrations, these metals had noticeable effects on the macrobenthic community. Heavy metals in seawater had a more substantial impact on the macrobenthic community than those in sediments, with Cr in seawater having the most significant influence on the community. These findings suggest that more attention should be given to Cr levels in western coastal waters of South Korea and that current seawater quality standards might need to be reconsidered to better reflect ecological risks posed by Cr, even at concentrations below regulatory limits.

## Figures and Tables

**Table 1:** Seawater environment data in subtidal zones of Asan Bay.

Environment Data	Range (Min–Max)	Mean ± SD	CV
Seawater temperature, °C	1.30–26.20	18.18 ± 8.56	0.47
Salinity, PSU	27.65–30.50	29.26 ± 0.94	0.03
pH	7.77–8.14	8.01 ± 0.09	0.01
DO, mg/L	6.92–12.95	8.36 ± 1.67	0.20
COD, mg/L	1.20–2.43	1.55 ± 0.31	0.20
Total Nitrogen, mg/L	0.44–1.42	0.91 ± 0.33	0.36
Total Phosphorus, mg/L	0.03–0.09	0.06 ± 0.02	0.25
Suspended Solids, mg/L	10.40–36.60	19.99 ± 5.49	0.27
As, μg/L	0.13–0.89	0.33 ± 0.17	0.51
Cd, μg/L	0.13–0.44	0.27 ± 0.08	0.29
Cr, μg/L	0.05–0.68	0.25 ± 0.20	0.80
Cu, μg/L	0.36–4.30	1.76 ± 1.17	0.67
Pb, μg/L	0.16–1.94	0.60 ± 0.35	0.59
Zn, μg/L	0.76–6.46	2.86 ± 1.55	0.54
Hg, μg/L	0–0.01	0.002 ± 0	0.66

Note: COD, chemical oxygen demand; DO, dissolved oxygen; CV, Coefficient of variation.

**Table 2:** Sediment environment data in subtidal zones of Asan Bay.

Environment Data	Range (Min–Max)	Mean ± SD	CV
AVS, mg/g	0.02–0.12	0.06 ± 0.02	0.35
COD, mg/kg	2.37–14.00	5.54 ± 2.51	0.45
IL, %	1.55–8.14	3.92 ± 1.67	0.43
Mean grain size, ∮	2.30–8.10	5.01 ± 1.77	0.35
As, mg/kg	0.13–0.64	0.35 ± 0.12	0.35
Cd, mg/kg	0.47–0.98	0.71 ± 0.11	0.15
Cr, mg/kg	2.03–22.82	11.39 ± 4.31	0.38
Cu, mg/kg	5.01–13.54	9.02 ± 2.04	0.23
Pb, mg/kg	12.47–33.20	21.71 ± 4.83	0.22
Zn, mg/kg	18.48–65.07	46.12 ± 10.37	0.22
Hg, mg/kg	0–0.01	0.003 ± 0	0.38

Note: AVS, acid-volatile sulfide; COD, chemical oxygen demand; IL, ignition loss; CV, Coefficient of variation; SD, Standard deviation.

**Table 3:** Dominant species in subtidal zones of Asan Bay.

Taxa	Specie	Dominant Value
Echinodermata	*Amphiodia craterodmeta*	0.20
Annelida	*Heteromastus filiformis*	0.17
Annelida	*Ampharete finmarchica*	0.10
Arthropoda	*Corophium* sp.	0.03

**Table 4:** DistLM results revealing influence of environmental data on macrobenthic community structure. Significance levels were determined by permutation tests (999 permutations).

Environment Data	Pseudo -F	P	Proportion of Variation Explained
Seawater temperature, °C	3.17	0.002	0.05
As in seawater, μg/L	4.04	0.001	0.07
Cr in seawater, μg/L	5.33	0.001	0.07
Cu in seawater, μg/L	5.35	0.001	0.08
Pb in seawater, μg/L	4.20	0.001	0.07
Hg in seawater, μg/L	2.41	0.006	0.04
Ignition loss, %	0.69	0.780	0.01
Cr in sediment, mg/kg	1.25	0.205	0.02
Hg in sediment, mg/kg	1.63	0.067	0.03

## Data Availability

The raw data supporting the conclusions of this article will be made available by the authors on request.

## Supplementary Materials

The following supporting information can be downloaded at: https://www.mdpi.com/article/10.3390/biology14091276/s1, Figure S1. Values of Species richness index (d) at each sampling site; Figure S2. Values of Pielou’s evenness index(J’) at each sampling site; Figure S3. Values of Shannon-Wiener diversity index (H’) at each sampling site; Figure S4. Values of Simpson index (1-Lambda’) at each sampling site; Figure S5. Geographic locations of the study area (Asan Bay) and comparison sites (Gyeonggi Bay, Masan Bay, Jindo Island, and Jeju Island) along the Korean coast, including heavy metal concentrations in seawater; Figure S6. Geographic locations of the study area (Asan Bay) and comparison sites (Dangdong Bay, South sea of Korea, and East sea of Korea) along the Korean coast, including heavy metal concentrations in sediment; Table S1. Coordinates of sampling stations; Table S2. The formulae for calculating the dominance and ecological indices; Table S3. Eigenvectors of seawater environmental factors with PC1 and PC2; Table S4. Eigenvectors of sediment environmental factors with PC1 and PC2; Table S5. RDA results revealed the influence of environmental data on the macrobenthos; Table S6. Eigenvectors of environmental factors to the dbRDA axes 1 and 2; Table S7. The five best results from BIO-ENV analyses for the subtidal zones of Asan Bay.
