# Supplementary material for: Responses of Macrobenthic Communities to Heavy Metal Contamination in Sediments and Seawater: A Case Study in Temperate Bay, South Korea
Source: Biology (Basel). 2025 Sep 16;14(9):1276. doi: 10.3390/biology14091276 (PMC12467039; doi:10.3390/biology14091276)
Supplement: Supplementary file 1 [file biology-14-01276-s001.zip › biology-3856431-supplementary.pdf]

Supplementary Material

Responses of Macrobenthic Communities to Heavy Metal Contamination in Sediments and Seawater: A Case Study in Temperate Bay, South Korea

Jian Liang[1,†], Se-Hyun Choi[2, 3,†], and Chae-Woo Ma[3, *]

[1] Department of Food Science and Engineering, Xinjiang Institute of Technology, Aksu 843000, People's Republic of China

[2] Fisheries Business Team, Korea Fisheries Infrastructure Public Agency, Seoul, Republic of Korea

[3] Department of Biology, College of Natural Sciences, Soonchunhyang University, Asan 31538, Republic of Korea

[†] These authors contributed as co-first authors.

[*] Correspondence: cwooma@sch.ac.kr

Contents

Table S1. Coordinates of sampling stations...2

Table S2. The formulae for calculating the dominance and ecological indices. 3

Table S3. Eigenvectors of seawater environmental factors with PC1 and PC2. 4

Table S4. Eigenvectors of sediment environmental factors with PC1 and PC2. 5

Table S5. RDA results revealed the influence of environmental data on the macrobenthos. 6

Table S6. Eigenvectors of seawater environmental factors to the dbRDA axes 1 and 2. 7

Table S7. The five best results from BIO-ENV analyses for the subtidal zones of Asan Bay. 8

Figure S1. Values of Species richness index (d) at each sampling site. 9

Figure S2. Values of Pielou's evenness index(J') at each sampling site..10

Figure S3. Values of Shannon-Wiener diversity index (H') at each sampling site. 11

Figure S4. Values of Simpson index (1-Lambda') at each sampling site..12

Figure S5. Geographical locations of the study area (Asan Bay) and the comparison sites (Gyeonggi Bay, Masan Bay, Jindo, and Jeju Island) along the Korean coast, including heavy metal concentrations in seawater.. 13

Figure S6. Geographic locations of the study area (Asan Bay) and comparison sites (Dangdong Bay, South sea of Korea, and East sea of Korea) along the Korean coast, including heavy metal concentrations in sediment. 14

Table S1. Coordinates of sampling stations.

Sampling site	Latitude	Longitude
S1	37°00'03.03"N	126°42'22.01"E
S2	36°59'45.13"N	126°43'28.52"E
S3	36°59'32.70"N	126°44'35.01"E
S4	36°59'32.06"N	126°46'11.02"E
S5	37°00'48.54"N	126°45'01.99"E
S6	37°00'02.02"N	126°44'37.09"E
S7	37°00'28.02"N	126°43'58.01"E
S8	37°00'31.01"N	126°43'13.04"E
S9	37°01'21.02"N	126°43'07.63"E
S10	37°00'53.99"N	126°41'51.06"E

Table S2. The formulae for calculating the dominance and ecological indices.

Indices	Algorithm	Note
Dominance index (Y)	= (ni/N) × fi,	'N' denoted the total number of individuals across all species, 'ni' was the number of individuals of the ith species, and 'fi'was the occurrence frequency of the ith species at study area
Species richness index (d)	$= (S - 1)/\log(\mathrm{N})$	S: The total number of species; N: The number of individual organisms.
Pielou's evenness index(J')	$= H'/\log(\mathrm{S})$	H': Shannon-Wiener diversity index; S: The total number of species.
Simpson index (1-Lambda')	$= 1 - \mathrm{SUM(Ni^*(Ni-1)/(N^*(N-1))}$	Ni: Number of individuals of the ith species; N: The number of individual organisms.
Shannon-Wiener diversity index (H')	$= -\sum\left[\left(\frac{n_i}{N}\right)\log_2\left(\frac{n_i}{N}\right)\right]$	Ni: Number of individuals belonging to the ith species; N: Total number of individuals.

Table S3. Eigenvectors of seawater environmental factors with PC1 and PC2.

Environment Factors	PC1	PC2
Seawater temperature, °C	0.178	-0.113
Salinity, PSU	-0.287	0.092
pH	-0.056	-0.222
DO, mg/L	-0.144	0.083
COD, mg/L	-0.217	-0.153
Total Nitrogen, mg/L	0.286	0.033
Total Phosphorus, mg/L	0.270	-0.043
Suspended Solids, mg/L	0.232	-0.093
As, μg/L	0.247	0.026
Cd, μg/L	0.219	-0.036
Cr, μg/L	0.312	-0.105
Cu, μg/L	-0.309	-0.093
Pb, μg/L	-0.243	0.188
Zn, μg/L	-0.267	0.179
Hg, μg/L	0.132	0.324

Note: COD, chemical oxygen demand; DO, dissolved oxygen.

Table S4. Eigenvectors of sediment environmental factors with PC1 and PC2.

**Environment Factors**	**PC1**	**PC2**
AVS, mg/g	-0.274	-0.094
COD, mg/Kg	-0.208	-0.177
IL, %	-0.099	-0.246
Mean grain size, ∮	0.010	-0.473
As, mg/kg	0.059	0.347
Cd, mg/kg	0.004	0.243
Cr, mg/kg	-0.062	0.297
Cu, mg/kg	-0.107	-0.136
Pb, mg/kg	0.092	0.017
Zn, mg/kg	-0.044	0.127
Hg, mg/kg	-0.010	0.259

Note: AVS, acid-volatile sulfide; COD, chemical oxygen demand; DO, dissolved oxygen; IL, ignition loss.

Table S5. RDA results revealed the influence of environmental data on the macrobenthos.

Environment data	R^2	Sum of Squares	Pseudo -F	P	Contribution
Seawater temperature, ℃	0.05	7099	0.6	0.63	2.7
As in seawater, μg /L	0.19	2377	1.9	0.104	9.6
Cr in seawater, μg /L	0.26	3983	9.3	0.002	47.6
Cu in seawater, μg/L	0.17	1937	0.3	0.3	1.7
Pb in seawater, μg /L	0.11	7290	1.8	0.118	8.6
Hg in seawater, μg/L	0.21	2945	1.2	0.264	5.8
Ignition loss, %	0.23	2302	1.8	0.136	9
Cr in sediment, mg/Kg	0.26	3983	2.1	0.086	10.8
Hg in sediment, mg/Kg	0.29	3950	0.9	0.448	4.2

Note: COD, chemical oxygen demand; DO, dissolved oxygen.

Table S6. Eigenvectors of seawater environmental factors to the dbRDA axes 1 and 2.

Environment Factors	dbRDA1	dbRDA2
Seawater temperature, ℃	-0.378	0.137
As in seawater, μg/L	-0.236	0.203
Cr in seawater, μg/L	-0.450	-0.259
Cu in seawater, μg/L	0.708	0.008
Pb in seawater, μg/L	0.180	-0.739
Hg in seawater, μg/L	-0.191	-0.182
Ignition loss, %	-0.102	-0.326
Cr in sediment, mg/Kg	-0.059	-0.407
Hg in sediment, mg/Kg	0.125	0.145

Table S7. The five best results from BIO-ENV analyses for the subtidal zones of Asan Bay.

Number of environmental variables	Correlation coefficient	Environmental variables
4	0.202	Temp, Pb-W, Cr-W, Cu-W
5	0.202	Temp, Pb-W, Cr-W, Cu-W, Hg-W
5	0.202	Temp, Pb-W, Cr-W, Cu-W, Hg-S
3	0.202	Temp, Pb-W, Cu-W
4	0.202	Temp, Pb-W, Cu-W, Hg-W

Note: DO, dissolved oxygen; S indicates sediment samples, and W indicates seawater samples.

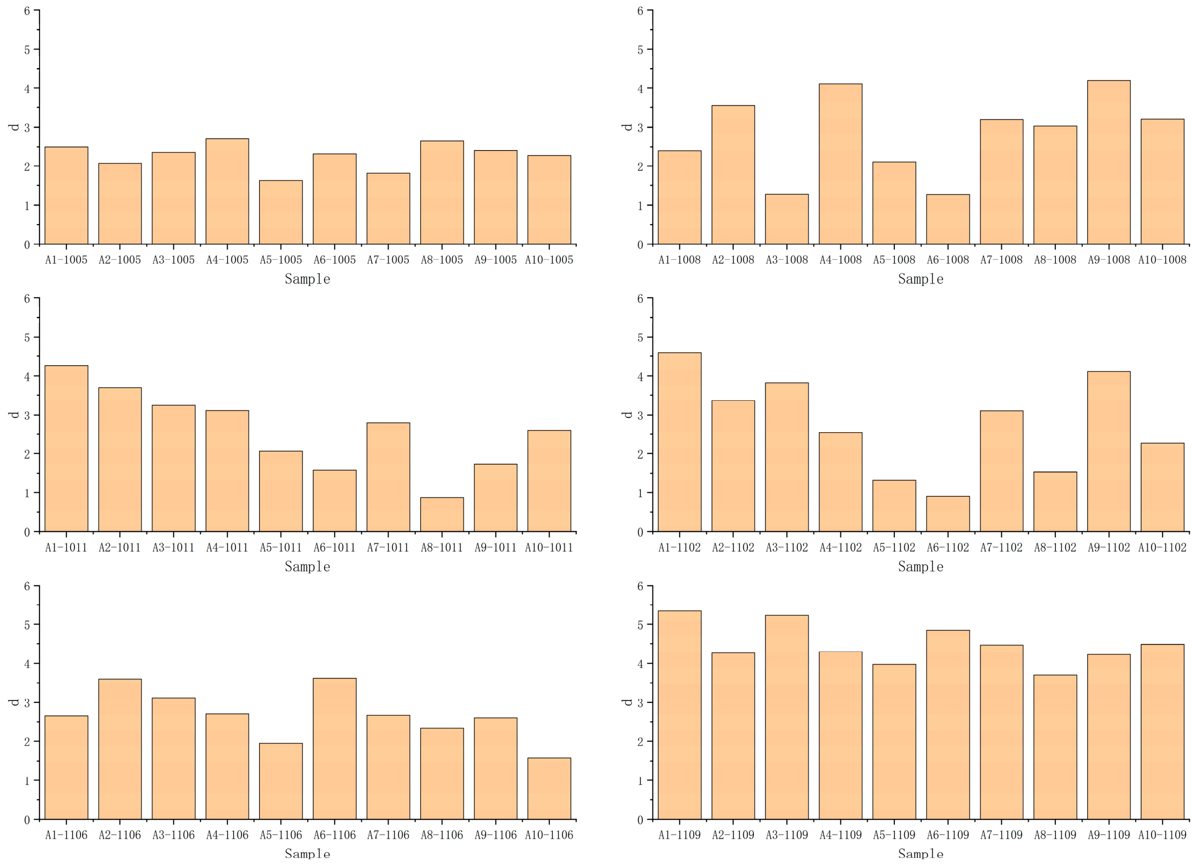

Figure S1. Values of Species richness index (d) at each sampling site.

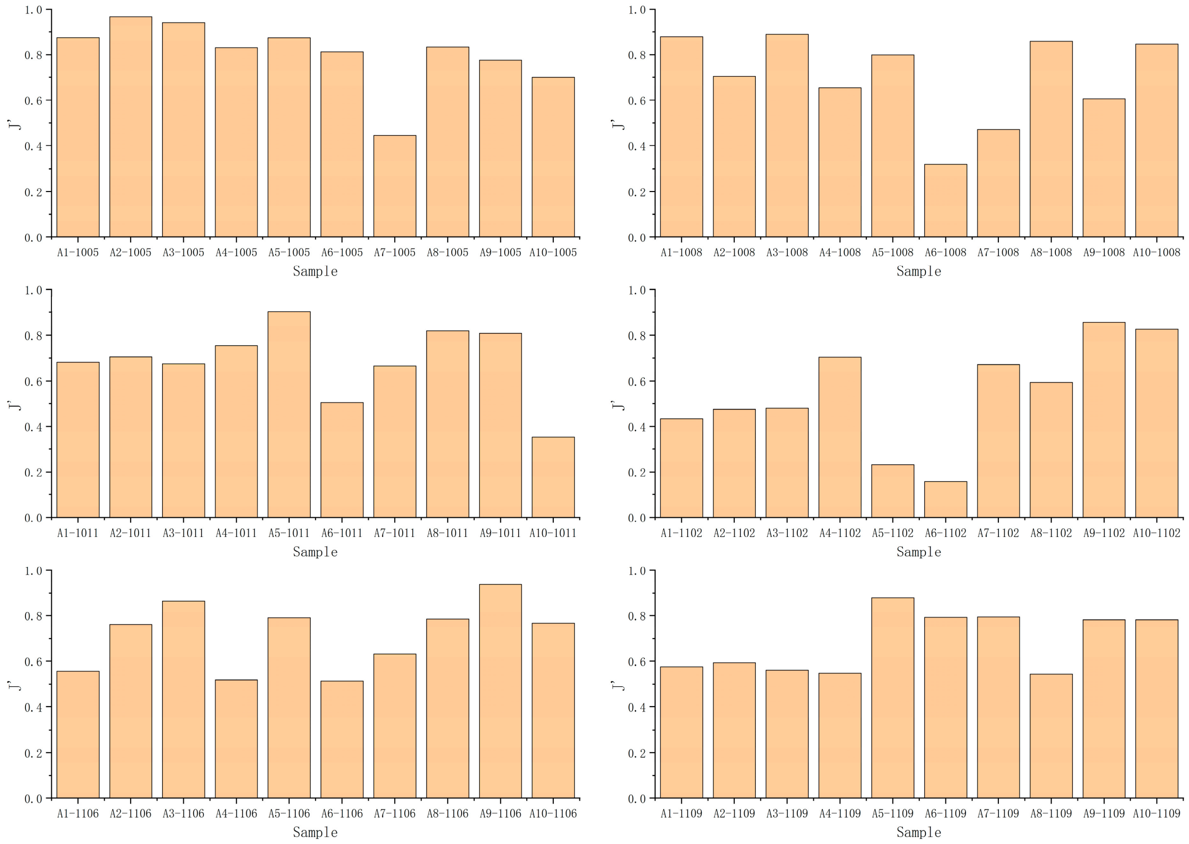

Figure S2. Values of Pielou's evenness index(J') at each sampling site.

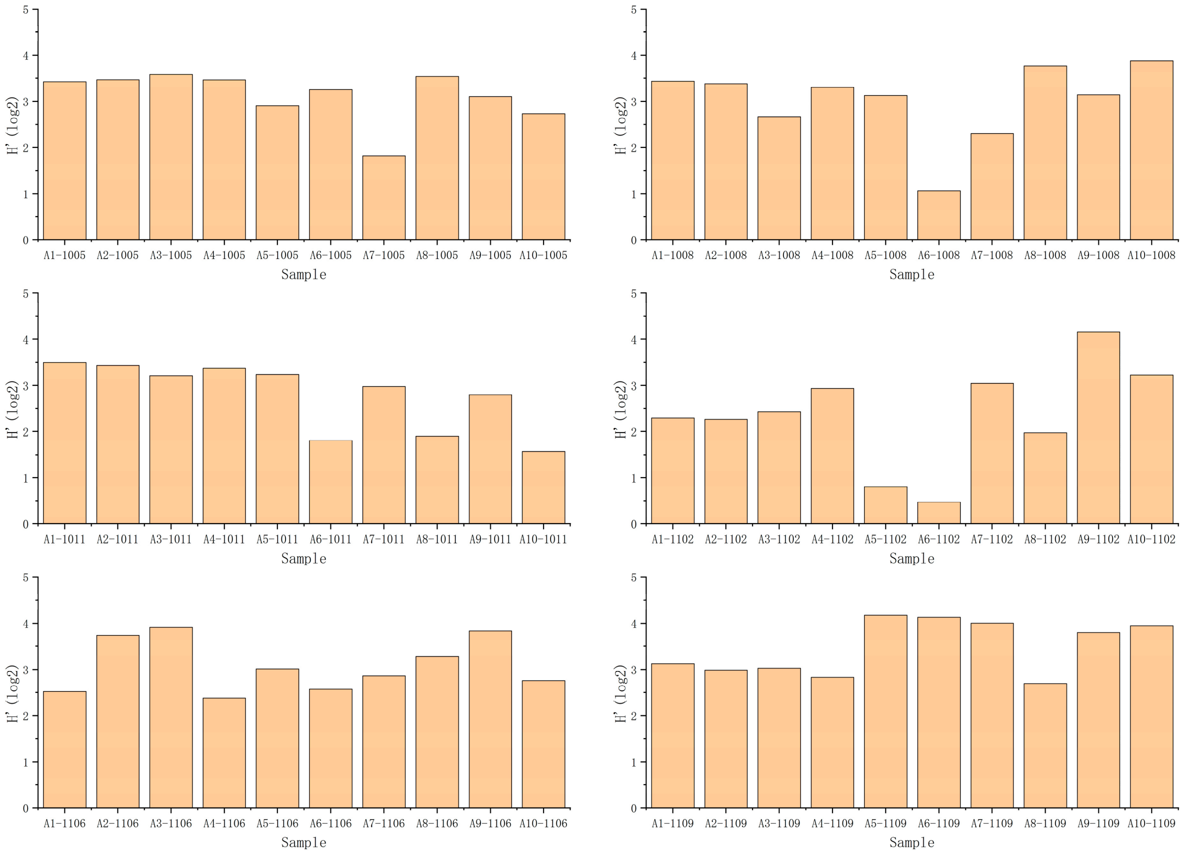

Figure S3. Values of Shannon-Wiener diversity index (H') at each sampling site.

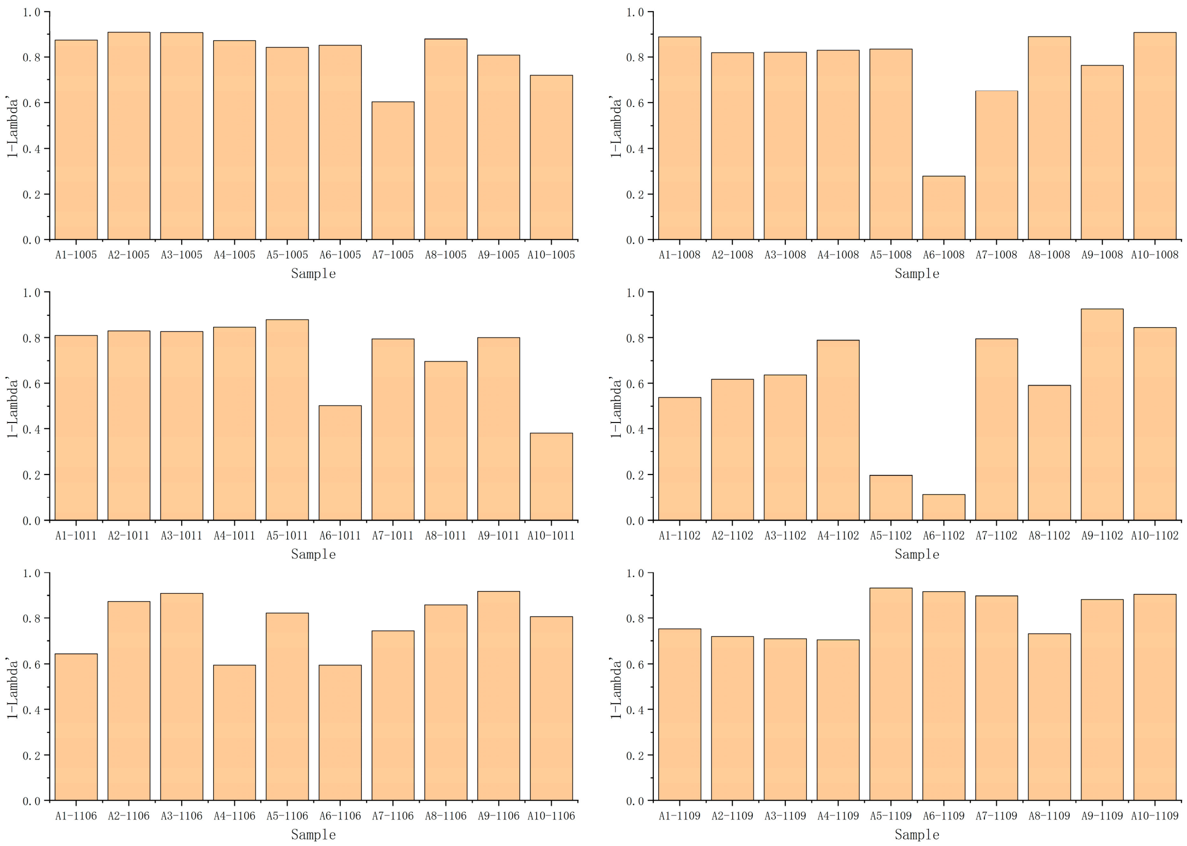

Figure S4. Values of Simpson index (1-Lambda') at each sampling site.

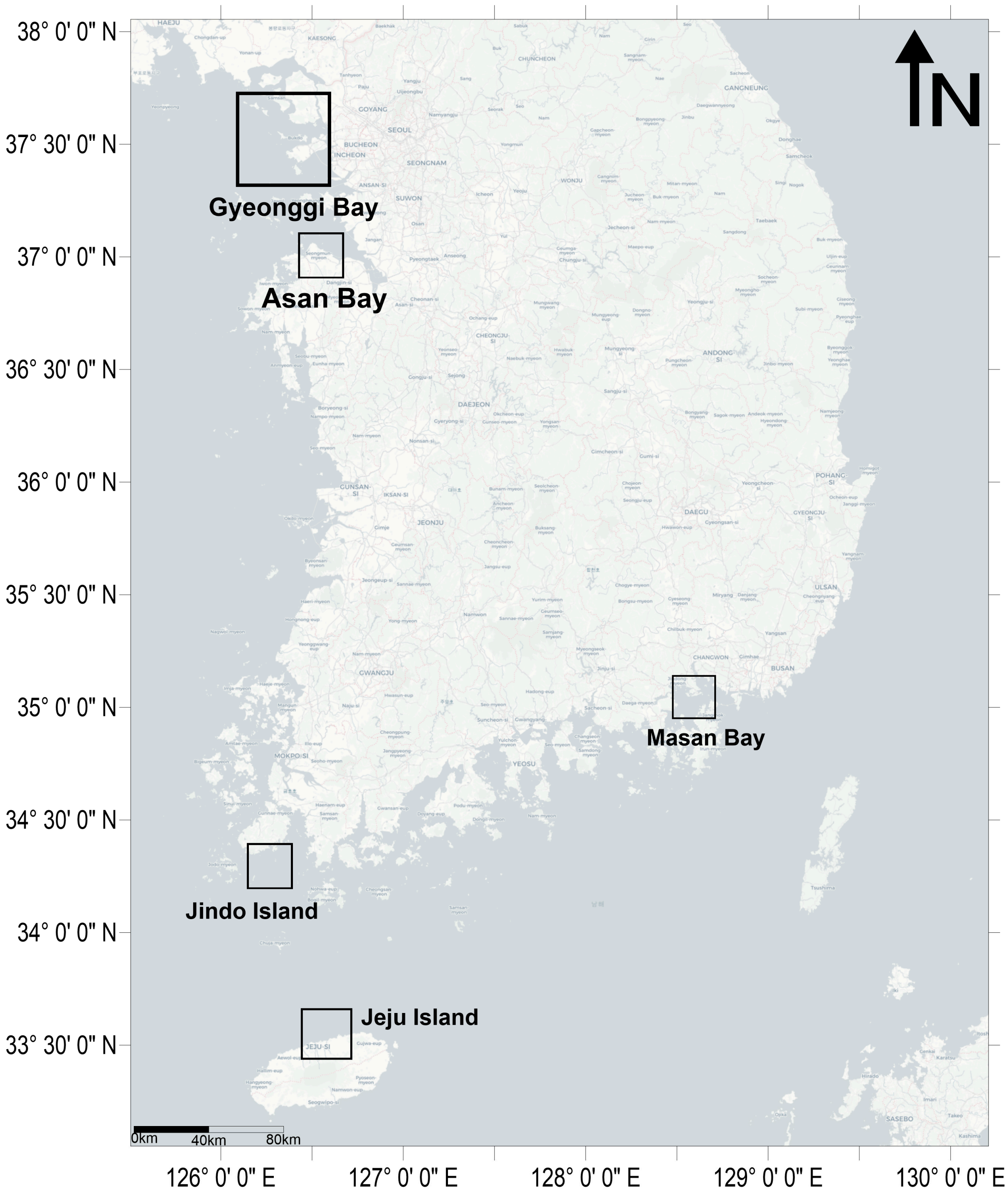

Figure S5. Geographical locations of the study area (Asan Bay) and the comparison sites (Gyeonggi Bay, Masan Bay, Jindo, and Jeju Island) along the Korean coast, including heavy metal concentrations in seawater.

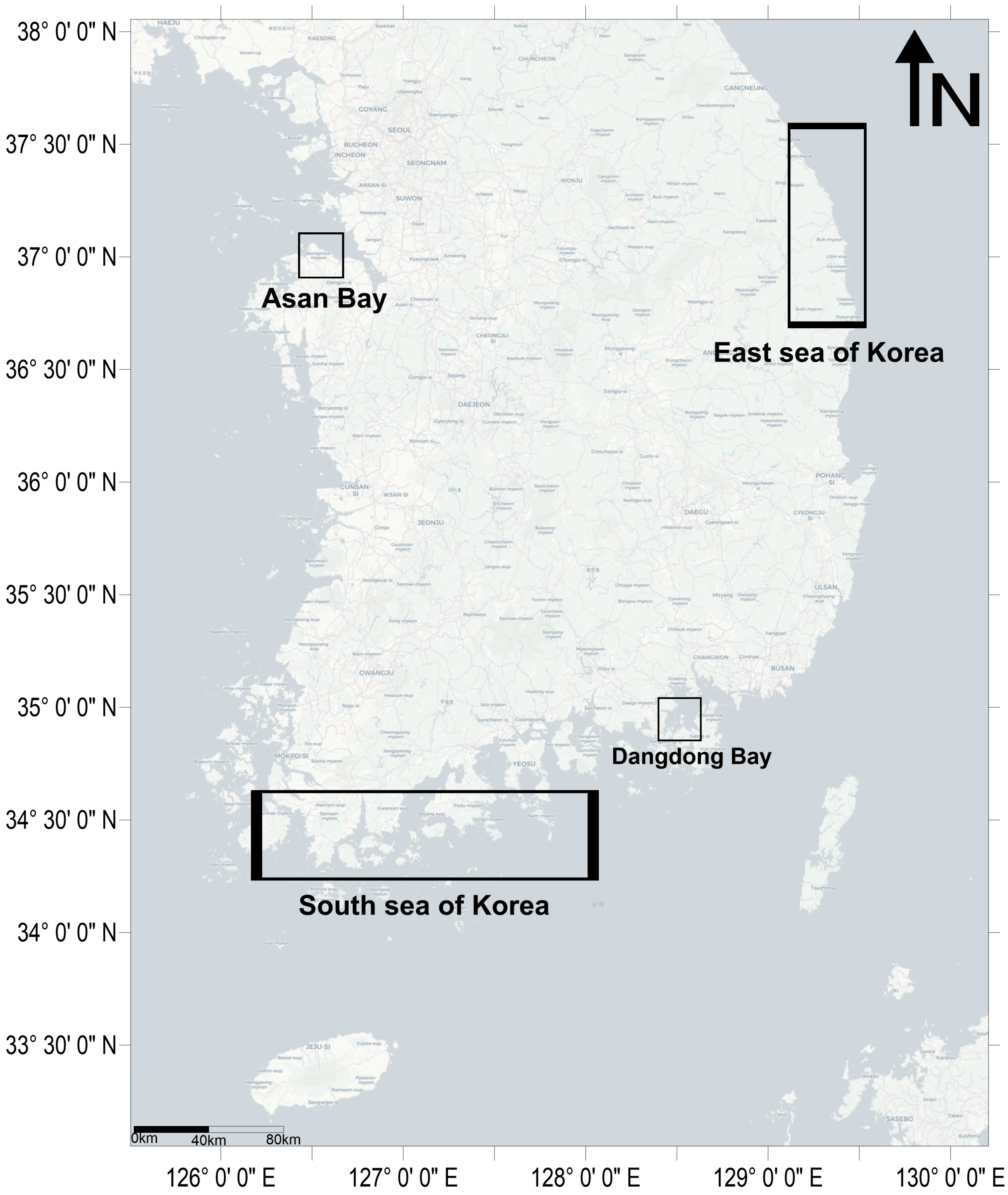

Figure S6. Geographic locations of the study area (Asan Bay) and comparison sites (Dangdong Bay, South sea of Korea, and East sea of Korea) along the Korean coast, including heavy metal concentrations in sediment.
